# Genome-wide Identification and Expression Analysis of *RcMYB* Genes in *Rhodiola crenulata*

**DOI:** 10.3389/fgene.2022.831611

**Published:** 2022-03-31

**Authors:** Binjie Xu, Bang Chen, Xiaoli Qi, Shunli Liu, Yibing Zhao, Ce Tang, Xianli Meng

**Affiliations:** ^1^ Key Laboratory of Southwestern Chinese Medicine Resources, Innovative Institute of Chinese Medicine and Pharmacy, Chengdu University of Traditional Chinese Medicine, Chengdu, China; ^2^ China Resources Sanjiu (Ya’an) Pharmaceutical Co., Ltd., Ya’an, China; ^3^ School of Medical Technology, Chengdu University of Traditional Chinese Medicine, Chengdu, China

**Keywords:** Rhodiola crenulata, MYB transcription factors family, genome-wide identification, gene expression analysis, WGCNA analysis

## Abstract

Modern research has proved that the main medicinal component of *Rhodiola crenulata*, which has a wide range of medicinal value, is its secondary metabolite salidroside. The MYB transcription factor family is widely involved in biosynthesis of second metabolism and other roles in the stress response in plants, so a genome-wide identification and analysis for this family in *R. crenulata* is worth conducting. In this research, genome-wide analysis identified 139 *MYB* genes based on conserved domains in the *R. crenulata* genome, and 137 genes were used to construct a phylogenetic tree and modified with expression files to reveal evolutionary characteristics. Physical and chemical characteristics, gene structure, and conserved motif analysis were also used to further analyze *RcMYBs*. Additionally, *cis*-acting elements related to transcription, hormone, and MYB binding were found in the promoter region of the selected *RcMYBs*. Four *RcMYBs* were cloned, sequenced, and their gene expression pattern was analyzed for further analysis of their functions. The research results lay the foundation for further research on the function of *RcMYB* and *R. crenulata*.

## Introduction

*Rhodiola* is a perennial plant in the genus *Rhodiola* of the Crassulaceae family, and is composed of more than 90 species. Most *Rhodiola* species grow in high-altitude and cold regions from Eurasia to North America. Among them, 73 varieties of *Rhodiola* are distributed in China. *Rhodiola* extract has also shown anti-inflammatory and anti-radiation effects and prevent cardiovascular diseases and cancer (Qu et al., 2012; Fan et al., 2020). Salidroside, which is mainly found in roots and stems, is the most medicinal functional ingredient in *Rhodiola*, and its content is an important factor in evaluating the quality of *Rhodiola*. Previous studies have shown that the biosynthesis of salidroside is derived from tyrosine, and some enzymes, tyrosine decarboxylase (TyDC), monoamine oxidase (MAO), 4-hydroxyphenylacetaldehyde reductase (4HPAR), UGTs, and 4-HPAA synthase (4HPAAS), are involved in the pathway (Lan et al., 2013; Torrens-Spence et al., 2017).

MYB transcription factors are one of the largest transcription factor family in plants, with the characteristics of multiple species and diversified functions (Prouse and Campbell, 2012), especially playing an important role in the regulation of plant secondary metabolism, such as the synthesis of flavonoids (Lai et al., 2013; Liu et al., 2015). It is found that MYB-conserved domains form a helix-turn-helix (HTH) structure in space and the structure interacts with regulatory elements in the promoter. The number and position of repeated MYB domains and MYB transcription factors are divided into four categories, namely 1R-MYB/MYB-related, R2R3-MYB, 3R-MYB, and 4R-MYB (Jiang et al., 2004a; Dubos et al., 2010). Among them, the R2R3-MYB protein is the most studied. Many members of this family have important regulatory effects in many biological processes, such as plant growth and development, organ morphogenesis, secondary metabolism, and stress response (Butt et al., 2017).

The research on *MYB* genes in plants mostly covers *Arabidopsis thaliana* and *Oryza sativa*. About 126 R2R3-MYB subfamily members have been identified in *Arabidopsis thaliana*, and their functions have been demonstrated and verified (Yanhui et al., 2006). These characterized *AtMYBs* can be used to predict the functions of MYBs in other species by homology analysis. So far, no research on the MYB transcription factor family in *R. crenulata* has been completed.

In this research, a total of 139 *RcMYB* genes of *R. crenulata* were identified using a known MYB genome sequence from *A. thaliana*. Moreover, the exon-intron structure, chromosome location, phylogenetic relationships, conserved motifs, and promotor elements of *RcMYB* genes were clarified. Besides, a co-expression network was constructed based on the expression patterns of key genes in the biosynthetic pathway of salidroside and the identified *MYB* genes, aiming to speculate on the *RcMYB* members involved in regulating the synthesis of salidroside. The selected *RcMYB* genes were verified by cloning and resequencing. Finally, the expression pattern of these selected *RcMYBs* in different tissues was determined. Based on the current data, this research is the first report on genome-wide gene family identification in *R. crenulata.* Our research may be helpful for further study about the functional characterization of *RcMYBs*.

## Materials and Methods

### Plant Materials

The tissue culture materials of *R. crenulata* used in the experiment were collected from unknown mountains in Wenchuan county, located in the northwest of Sichuan province, China (Supplementary Figure S1).

### Genome-wide Identification of *MYB* Genes in *R. crenulata*

To identify the *RcMYB* genes, the whole-genome sequencing result data of *R. crenulata* (Fu et al., 2017) from GigaDB (http://gigadb.org/dataset/10030) and annotation files from NCBI (https://www.ncbi. nlm.nih.gov/) were downloaded. Then, the candidate *RcMYB* sequences were screened with the online tool Pfam (http://pfam.xfam.org/) to identify the MYB domain, and sequences containing no conserved MYB domains were deleted. Further, BLAST comparison by submitting the identified *RcMYBs* into the NCBI database was performed (Ron et al., 2002). All *AtMYBs* were downloaded from PlantTFDB v5.0 (http://planttfdb.gao-lab.org/), along with the function annotation from the *Arabidopsis* Information Resource (TAIR) (https://www.arabidopsis.org/) and NCBI databases. The secondary metabolism-related *AtMYBs* were selected as the comparison group for subsequent screening of target *RcMYBs*.

### Chromosomal Location of *RcMYB* Genes and Assessment of Genome Quality

Gene ID information of the *RcMYB* loci on the chromosome (scaffolds) was obtained from the genome annotation files (Fu et al., 2017). The distribution of *RcMYBs* on the chromosome (scaffolds) was generated with TBtools (Chen et al., 2020). The gene density was calculated and outputted by TBtools, while the GC density was calculated by Biostrings (https://bioconductor.org/packages/Biostrings). Analyzed results and annotations were visualized by an online Circos drawing (Gu et al., 2014).

### Multiple Sequence Alignment, Orthologous Gene Identification, and Phylogenetic Analysis

The phylogenetic analysis of RcMYBs was conducted using a method from a previous work (Long et al., 2021). Briefly, amino sequences of RcMYBs were aligned by Clustal W with default parameters and then a phylogenetic tree was constructed using the neighbor joining (NJ) method in MEGA X after a selection of a substitution model (Kumar et al., 2018). The parameters of NJ analysis were as follows: bootstrap method done 1,000 times for statistical testing, model with JTT + G, and 50% sites coverage cutoff. The phylogenetic tree was modified by online software Interactive Tree Of Life (iTOLv6).

The physical and chemical characteristics of RcMYB proteins like molecular weight (MW) and isoelectric point (pI) were calculated by the ProtParam tool in the ExPASy Server (https://web.expasy.org/protparam/). All the amino acid sequences of RcMYB proteins were submitted to the online tool MEME suite (http://meme-suite.org/tools/meme) where we performed motifs analysis. Conserved motifs were detected with setting the repeat number to any (anr), the maximum number of motifs to 12, and the rest of the run parameters to system default. The structure diagram and LOGO of each motif from the result were downloaded. TBtools was used to combine the motif structure diagram with the phylogenetic tree, then a gene structure diagram of *RcMYBs* was outputted (Chen et al., 2020).

To explore the *cis*-elements in the promoter region of the *RcMYB* genes, 2 kb upstream *RcMYBs* sequences were uploaded to the PlantCARE database (http://bioinformatics.psb.ugent.be/webtools/plantcare/html/).

### Analysis of the Expression Profiles of *RcMYB* Genes

RNA-seq data in three tissues (root, stem, leaf) of *R. crenulata* were downloaded from NCBI respectively through SRA searching (SRX2577721 for root, SRX2577722 for stem, and SRX2577723 for leaf). We followed the method described by Wu and Zhang (2013). Briefly, transcriptome data were mapped to the *R. crenulata* genome by HISAT2 (version 2.2.1), and results were output in the SAM format. SAMtools (version 1.6) was used to convert result into the BAM format and we inputted the BAM file as a parameter into Stringtie (version 2.1.5) to generate the input file for Ballgown (version 2.22.0). Differential expression analysis was completed through software package Ballgown in R and visualized by iTOLv6 (https://itol.embl.de/).

### Weighted Gene Co-expression Network Construction and Hub Genes Identified

A gene co-expression network was constructed by using the WGCNA R package to further find *RcMYB* genes which might be involved in the metabolism of salidroside (Langfelder and Horvath, 2008). The FPKM values of 407 genes in three tissues were used to construct the network (Supplementary Table S1). The soft thresholding power β of nine was selected in order for the networks to exhibit an approximate scale-free topology. The adjacency matrix was transformed into a topological overlap matrix (TOM) to calculate the corresponding dissimilarity (1-TOM similarity). Afterward, a gene dendrogram was produced based on genes which were clustered using the TOM dissimilarity measure (Zhang and Horvath, 2005). Genes with co-expression patterns were grouped in the same module, and modules with salidroside metabolism pathway genes were visualized by Cytoscape (version 3.8.2; Shannon et al., 2003).

### Gene Cloning, Expression Analysis, and Vector Construction of Selected *RcMYB* Genes

DNA and RNA were extracted from the leaf and root tissues of *R. crenulata* individually. DNA extraction of materials was undertaken with CTAB methods (Minas et al., 2011), whereas RNA extraction followed the protocol of Trizol (TIANGEN, Beijing, China) reagent. RNA was reverse-transcribed using the PrimeScript™ RT Reagent Kit with a genomic DNA Eraser (Takara, Dalian, China), and quantitative RT-PCRs (qRT-PCR) were performed using a ChamQ SYBR qPCR Master Mix (Vazyme, Nanjing, China). All the experiments were performed following the manufacturer’s instructions. Data were normalized by the expression level of 18S rRNA (Lan et al., 2013), and the primers used for qRT-PCR are listed in Supplementary Table S2.

Four *RcMYB* candidate genes were isolated from different cDNAs using specific primers. The full-length cDNA sequence of the four *RcMYB* genes excluding the terminator codon were ampliﬁed by PCR. The PCR products were sub-cloned into the pEASY-Blunt cloning vector (TransGen Biotech, Beijing, China) and sequenced (Sangon Biotech, Shanghai, China). The primers used for vector contractor and sequencing are listed in Supplementary Table S2.

## Result

### Genome-wide Identification and Evolutionary Analysis of *RcMYB* Genes in *R. crenulata*

To identify *MYB* genes in the *R. crenulata* genome, the candidate *RcMYBs* were screened based on annotation and were uploaded to the Pfam34.0 database. Then two hidden Markov models (HMM) of MYB-DNA-binding domain (PF00249) and MYB-DNA-Bind 6 domain (PF13921) were used in the identified *RcMYBs* whereas the sequences without *MYB* domains were deleted. Identified *RcMYBs* were compared with *MYBs* in other species through NCBI BLAST for further verification. Finally, 139 *MYB* genes were confirmed from the *R. crenulata* reference genome, and then designated as *RcMYB1* to *RcMYB139* (Supplementary Table S6). Multiple sequence alignment of the *RcMYB*s was carried out by Clustal W, and showed that *RcMYB138* and *RcMYB139* had few common sites for alignment and were deleted from the phylogenetic tree. The length of most RcMYB protein sequences ranged from 150 to 550 amino acids (AA), and the theoretical isoelectric point (pI) ranged from 3.31 to 10.38 (Supplementary Table S6). The length of the two domains in *RcMYBs* were shown in Pfam: DNA-binding domain with a length of 46AA and DNA-bind 6 domain with a length of 60AA (Supplementary Figure S2A). Detailed information of *RcMYB* genes is shown in Supplementary Table S6.

**FIGURE 1:**
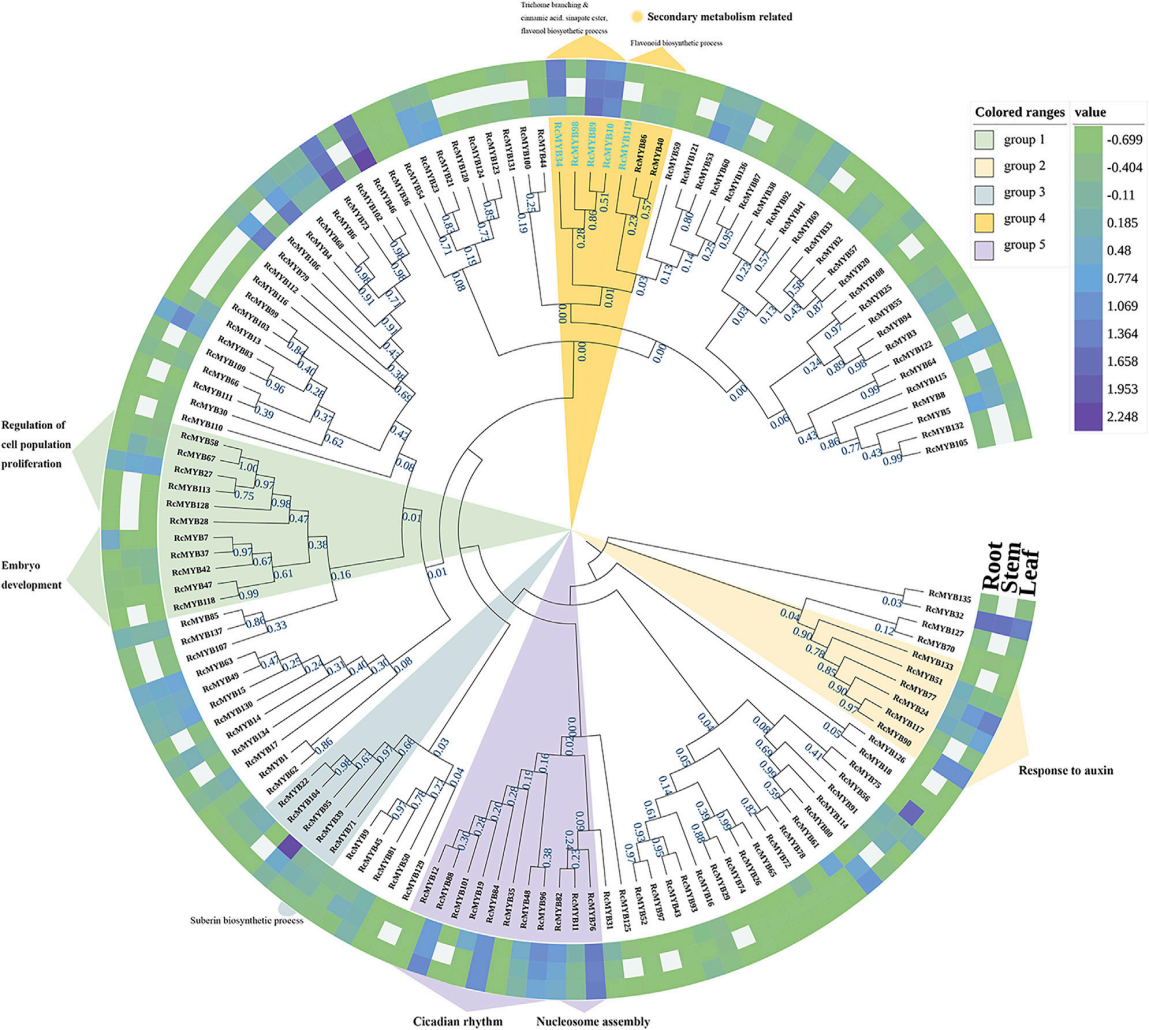
Phylogenetic tree of *RcMYB* genes with their relative expression levels in three tissues modified by iTOLv6.0. Functional annotations were predicted by BLAST in the TAIR database. Blank cells indicated data gaps and the labels of the selected *RcMYBs* were dyed blue. Value is equal to log_10_^(FPKM+0.2)^.

To figure out the phylogenetic relationship of *MYB* family genes, 137 *RcMYBs* were used to construct a neighbor joining phylogenetic tree by MEGA X (Figure 1). Gene expression level analysis needed to be added. To predict the function of RcMYBs, we selected the RcMYB protein with a relatively conserved sequence which was used as a BLASTP query in the TAIR database. Then genes clustered in five groups were predicted and marked by different colors. The *RcMYBs* in each group shared a high degree of similarity and were predicted to participate in certain biological processes. The *RcMYB* genes clustered in group 5 were conserved, though the BLAST results of *RcMYB11* and *RcMYB76* were extremely conservative in the process of nucleosome assembly, whereas the rest of the *RcMYBs* were predicted to mainly participate in circadian rhythm. Particularly, *RcMYB35* and *RcMYB84* were also speculated to be involved in the biosynthesis of auxin, while *RcMYB82* was thought to be involved in the biosynthesis of chlorophyll. Then we found that *RcMYB101* and *AtCCA1* have an extremely high sequence alignment score, which indicated that *RcMYB101* was very likely to be involved in the regulation of protein-containing complex assembly and response to organonitrogen compound, if *RcMYB101* retains the most primitive evolutionary information in this gene group (Eric et al., 2007; Wu and Zhang, 2013; Ye et al., 2016). The same situation also occurred in group 1 and the corresponding gene was *RcMYB28*, which showed a highly similarity to the left cluster of genes predicted to participate in embryo development and right cluster of genes involved in cell population proliferation, therefore it was predicted to participate in both processes in the database. The RcMYB genes clustered in group 2 were relatively conserved, and similar to *AtMYB96*, *AtMYB94*, and *AtMYB30*, were assumed to be involved in the response to auxin (Cui et al., 2016; Liao et al., 2017; Lee and Seo, 2019). The RcMYBs in group 3 may be involved in various biological processes through BLAST, such as wounding response, cell expansion, cutide deposition. Among them, *RcMYB71* was most likely to participate in suberin biosynthesis progress. The phylogenetic relationship revealed that RcMYBs in group 4 had extremely high comparison scores with the AtMYB proteins involved in secondary metabolism, such as cinnamic acid biosynthesis and flavonoid biosynthesis (Figure 1; Mehrtens et al., 2005; Liu et al., 2015).

### Gene Structure, Protein Motif, and Chromosomal Localization Analysis of *RcMYB* Genes in *R. crenulata*

**FIGURE 2:**
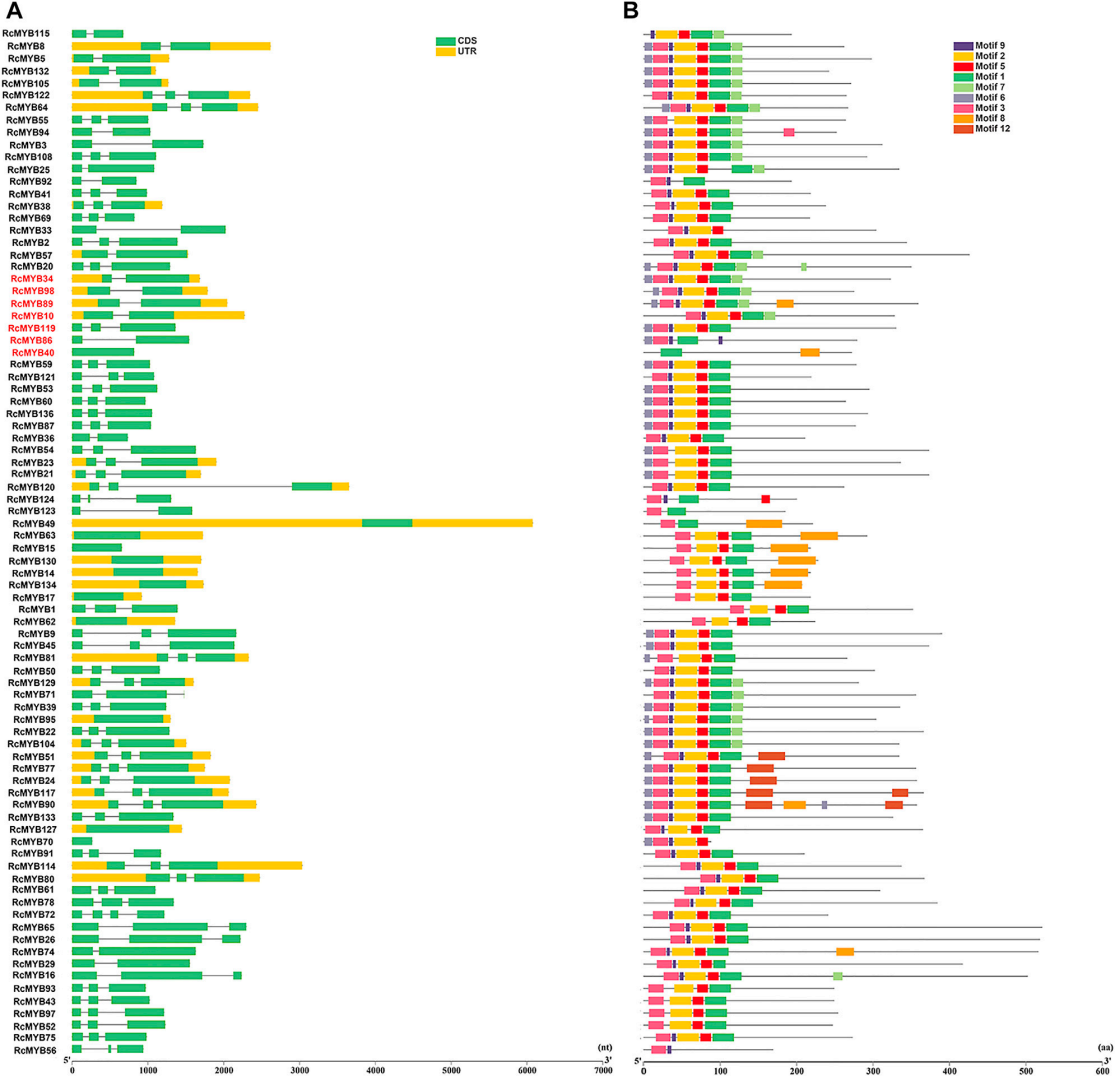
The gene structures and conserved motifs of R2R3 MYB proteins in *R. crenulata*. **(A)** The coding sequence (CDS) and untranslated region (UTR) were displayed in different colors, and the lines between boxes represented introns. **(B)** The phylogenetic tree constructed with RcMYB proteins and conserved domains of the RcMYB family was analyzed by MEME suite. Different colors indicated different motifs.

**FIGURE 3:**
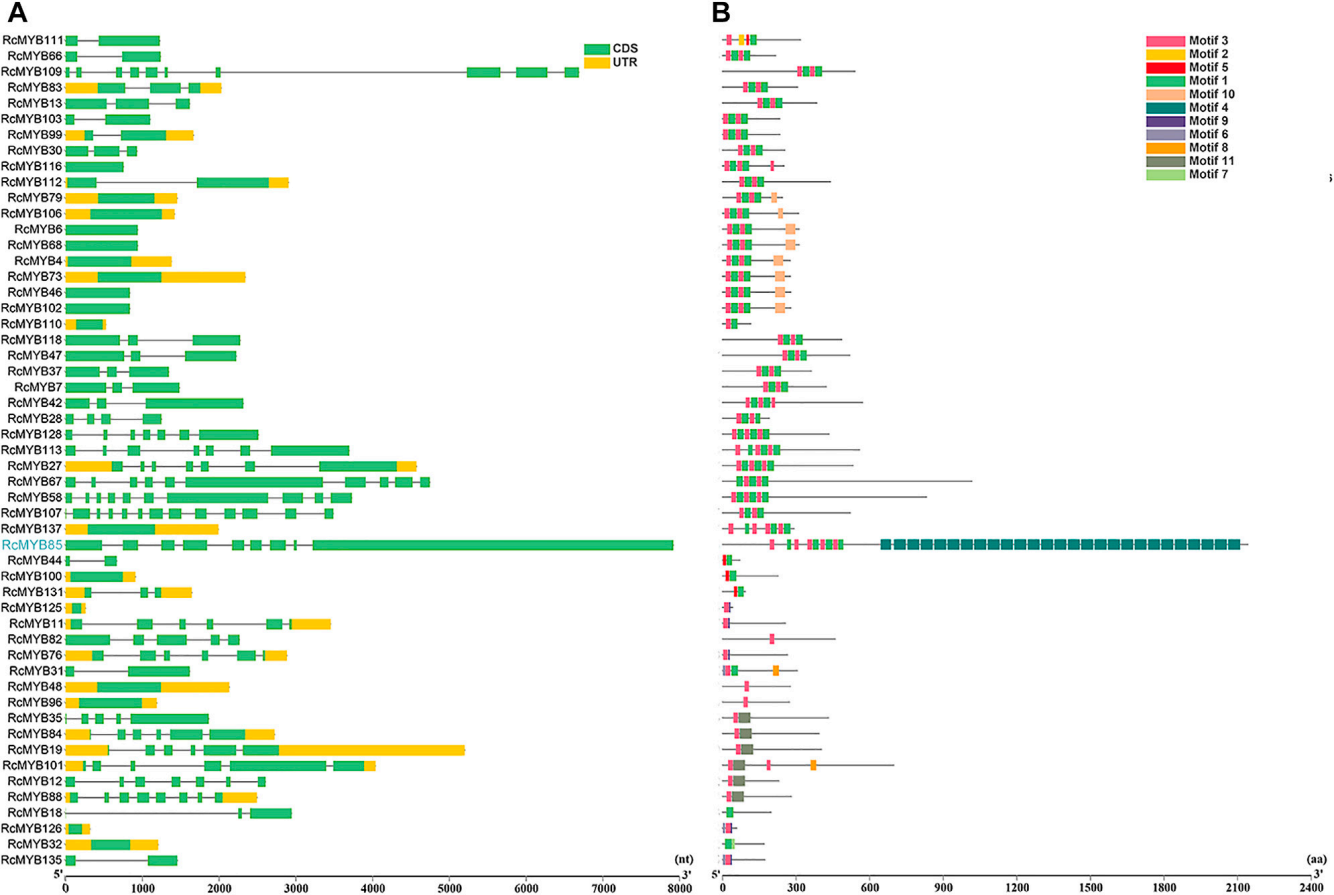
The gene structures and conserved motifs of *RcMYB* genes (1R-, 3R-, and 4R-) **(A)** The coding sequence (CDS) and untranslated region (UTR) were displayed in different colors, and the lines between boxes represented introns. **(B)** The phylogenetic tree constructed with RcMYB proteins and conserved domains of the RcMYB family was analyzed by MEME suite. Different colors indicated different motifs.

To explore the structural diversity of the *RcMYB* members, the exon-intron organization of each *RcMYB* gene was analyzed by comparing their cDNA sequences with the corresponding genomic DNA sequences. As shown in Figures 2A, 3A, most *RcMYB* genes contained one or two introns, whereas some *RcMYB* genes consisted of no introns. Particularly, *RcMYB107* contained 12 introns, while *RcMYB18* and *RcMYB120* contained a long intron in their gene structures. Notably, the gene structure of four genes (*RcMYB10*, *RcMYB34*, *RcMYB89*, and *RcMYB98*) clustered in group 4 showed a high similarity.

The features of *MYB* transcription factors were typically determined by their conserved motifs and domains. To have a more comprehensive understanding of the conserved domains of the *RcMYB* genes, motif elicitation (MEME) analysis was performed. That result showed that 12 conserved motifs of RcMYB proteins were predicted through the MEME suite (Figures 2B, 3B). In addition to what is shown in Figure 2B, specific amino acid sequences of each motif are also provided in Supplementary Figure S2B. Based on the number of the MYB domains and duplications of conserved sequences (Ambawat et al., 2013; Feng et al., 2017), MYB proteins in *R. crenulata* were classified into three types and as expected, R2R3-MYBs were in the majority (Supplementary Table S3). As shown in Figures 2B, 3B, motif 3 was found in most RcMYB proteins except RcMYB18, RcMYB32, RcMYB40, RcMYB44, RcMYB100, and RcMYB131. Interestingly, motif 4 in RcMYB85 repeated 27 times, which might suggest a unique function (Figure 3B). Some motifs like motif 8, motif 10, motif 11, and motif 12 were only found in genes clustered in the same group, indicating a similar function of these genes.

The evolutionary relationships within a gene family are typically analyzed according to their chromosomal distributions. Though the *R. crenulata* chromosomes were not assembled, we determined the corresponding scaffold information of the *RcMYB* genes based on the genome database. It was found that the *RcMYB* gene family was randomly distributed on 126 scaffolds, and only a few scaffolds contained multiple genes (Supplementary Figure S3). The exceptions were *RcMYB14* and *RcMYB15*, *RcMYB59* and *RcMYB60*, *RcMYB62* and *RcMYB63*, *RcMYB110* and *RcMYB111*, and *RcMYB123* and *RcMYB124*, which were located within 6 kb with each other on chromosomes, suggesting duplication events. To remove the influence of the high content of repetitive sequences in the draft genome, the 50 longest scaffolds with MYB-annotated genes were screened and identified. While the length of these scaffolds only accounted for 7.56% of the total length of scaffolds, the loaded structural genes size exceeded 26.18% of the total DNA size, which suggested that the genome was well-assembled (Supplementary Figure S4).

Considering the important of glycosides and flavonoids in *R. crenulata* for its medical use, *RcMYBs* involved in the secondary metabolism process, including *RcMYB10*, *RcMYB34*, *RcMYB89*, *RcMYB98,* and *RcMYB119*, were paid more attention (Figure 1). A phylogenetic tree was constructed by using the five *RcMYBs* with genes identified in tomato (*Solanum lycopersicum*), wheat (*Triticum aestivum*), *Arabidopsis* (*Arabidopsis thaliana*), and rice (*Oryza sativa*) that were involved in the secondary metabolism process (Matus et al., 2008; Ballester et al., 2010; Shen et al., 2019). The analysis showed that *RcMYB10*, *RcMYB34*, *RcMYB89*, and *RcMYB98* were clustered into the same group with *AtMYB5*, which functions in regulating flavonoid biosynthesis (Matus et al., 2008). *RcMYB119*, though, was clustered into the same group with *OsCYP93G1* and *Ta4CL2*; the similarity was not significant (Supplementary Figure S5).

**FIGURE 4:**
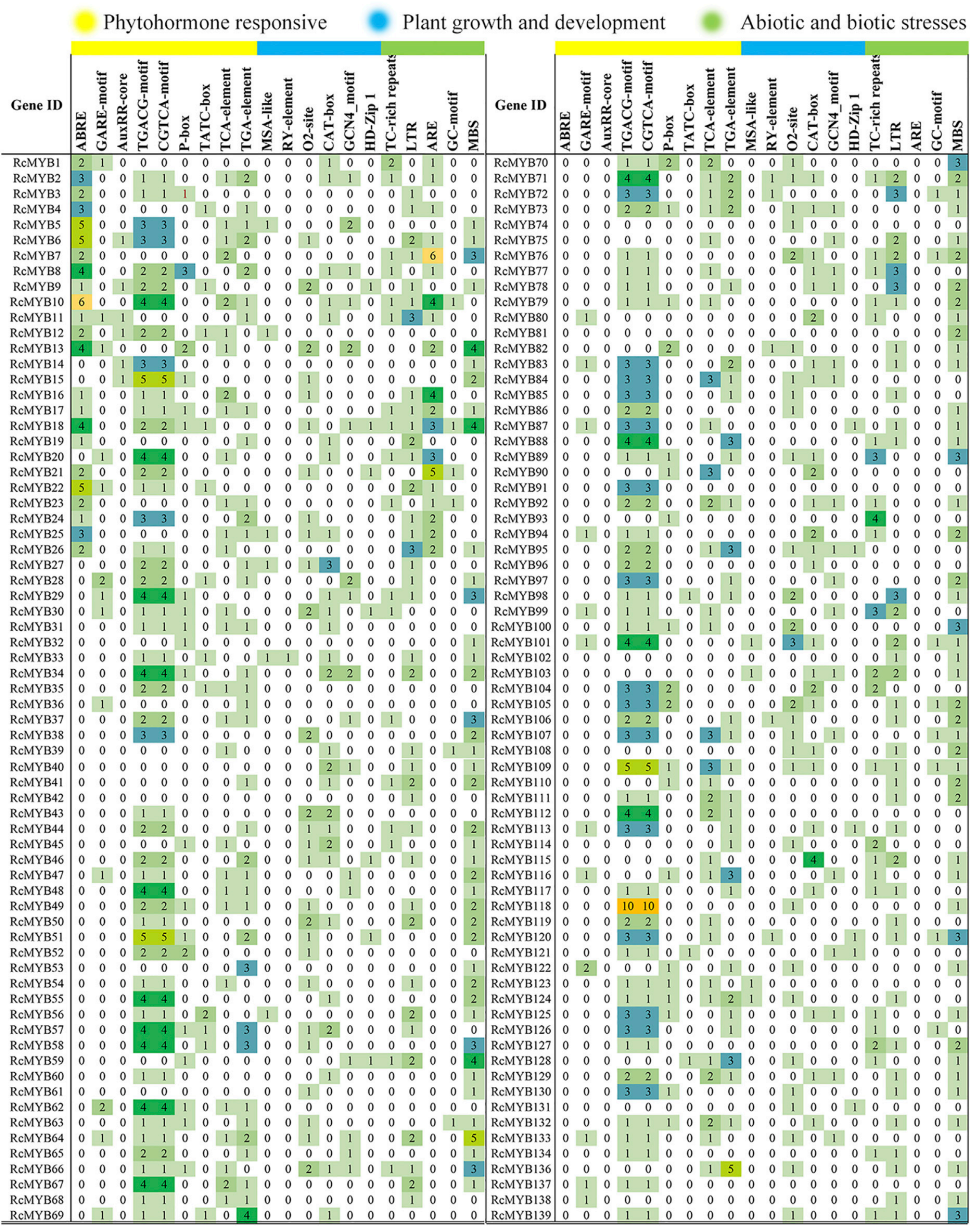
Number of each cis-acting element in the *RcMYB* genes in the promoter region. The detailed cis-element is listed in Supplementary Table S4.

To analyze the potential *cis*-elements, the 2 kb upstream sequences of the selected *RcMYB* genes were submitted to online tool PlantCARE. The type and position of *cis*-elements were marked in different colors (Figure 4), and their potential functions are annotated in Supplementary Table S4. Numerous *cis*-acting elements were detected and mainly divided into promoter element type and plant growth and environmental responsive type. Among these *cis*-elements, TATA-box, CAAT-box, and ABRE were conspicuous, which were involved in transcription initiation and abscisic acid response, indicating that these *RcMYB* genes are mainly involved in regulating the initiation of transcription and abiotic responses. The promoter region of *RcMYB118* contained 10 CGTCA-motifs and 10 TGACG-motifs, which are involved in MeJA-responsiveness, suggesting a hormone-regulating role of *RcMYB118*. The auxin-responsive element (Aux-RR core) were found in promotors of *RcMYB6*, *RcMYB9*, *RcMYB11*, *RcMYB12*, *RcMYB14,* and *RcMYB15*.

### Co-Expression Network Analysis of Candidate *RcMYB* Genes Involved in Salidroside Biosynthesis

**FIGURE 5:**
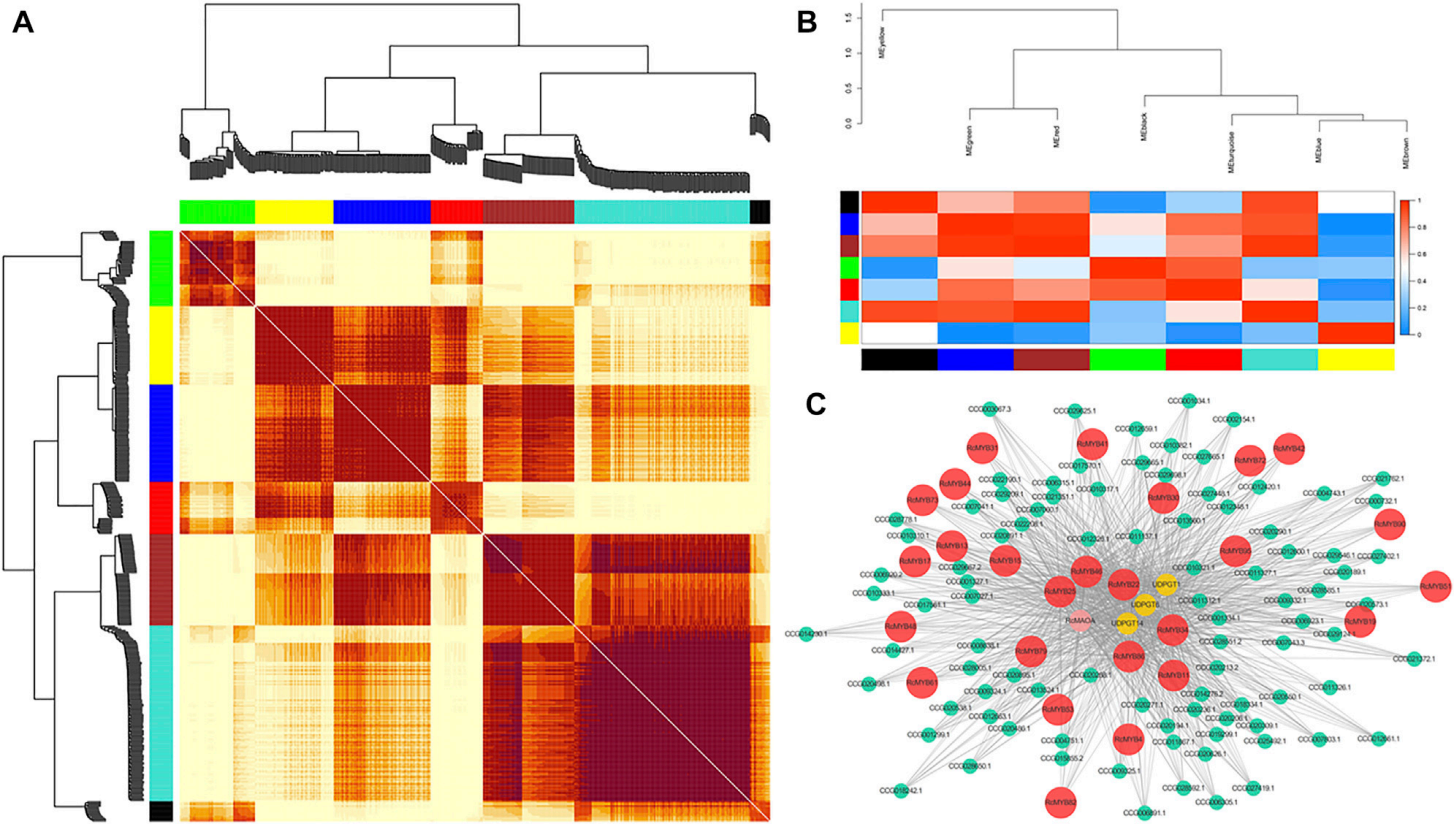
Co-expression network of *RcMYB* genes in *R. crenulata*. **(A)** The heatmap of correlation degree of modules. **(B)** Clustering of modules based on eigengenes. The colors ranging from blue to red represent Pearson correlation coefficients ranging from 0 to 1, indicating low to high correlations, respectively. **(C)** Visualization of connections of genes in the turquoise module. The *RcMYB* genes are colored in red. Connections of genes in other modules are shown in Supplementary Figure S6.

WGCNA analysis enabled the grouping of genes into seven co-expression networks (modules) with 407 genes (Figure 5A). The identification of the hub genes in each module was calculated by cytoHubba (version 0.1) through different algorithms, and the top 10 genes based on gene connection degrees were selected (Supplementary Table S5). These *RcMYBs* genes were selected as hub genes in each module and their connected genes were analyzed and visualized by Cytoscape to construct the network diagram (Figure 5C and Supplementary Figure S6). It was found that the expression pattern of *RcMYB10* showed a positive correlation with *RcTyDC9* (CCG026415.1) and *UDPGT4* (CCG024532.1), two genes encoding the key enzymes involved in salidroside biosynthesis (Supplementary Figure S6C). *RcMYB34*, however, showed a negative correlation with *UDPGT*s and *RcMAOA* (CCG009333.1*,* Figure 5C). This analysis suggested that *RcMYB10* and *RcMYB34* might play roles in regulating salidroside biosynthesis.

### Cloning and Expression Pattern Analysis of *RcMYB10*, *RcMYB34*, *RcMYB89,* and *RcMYB98* Genes in *R. crenulata*

**FIGURE 6:**
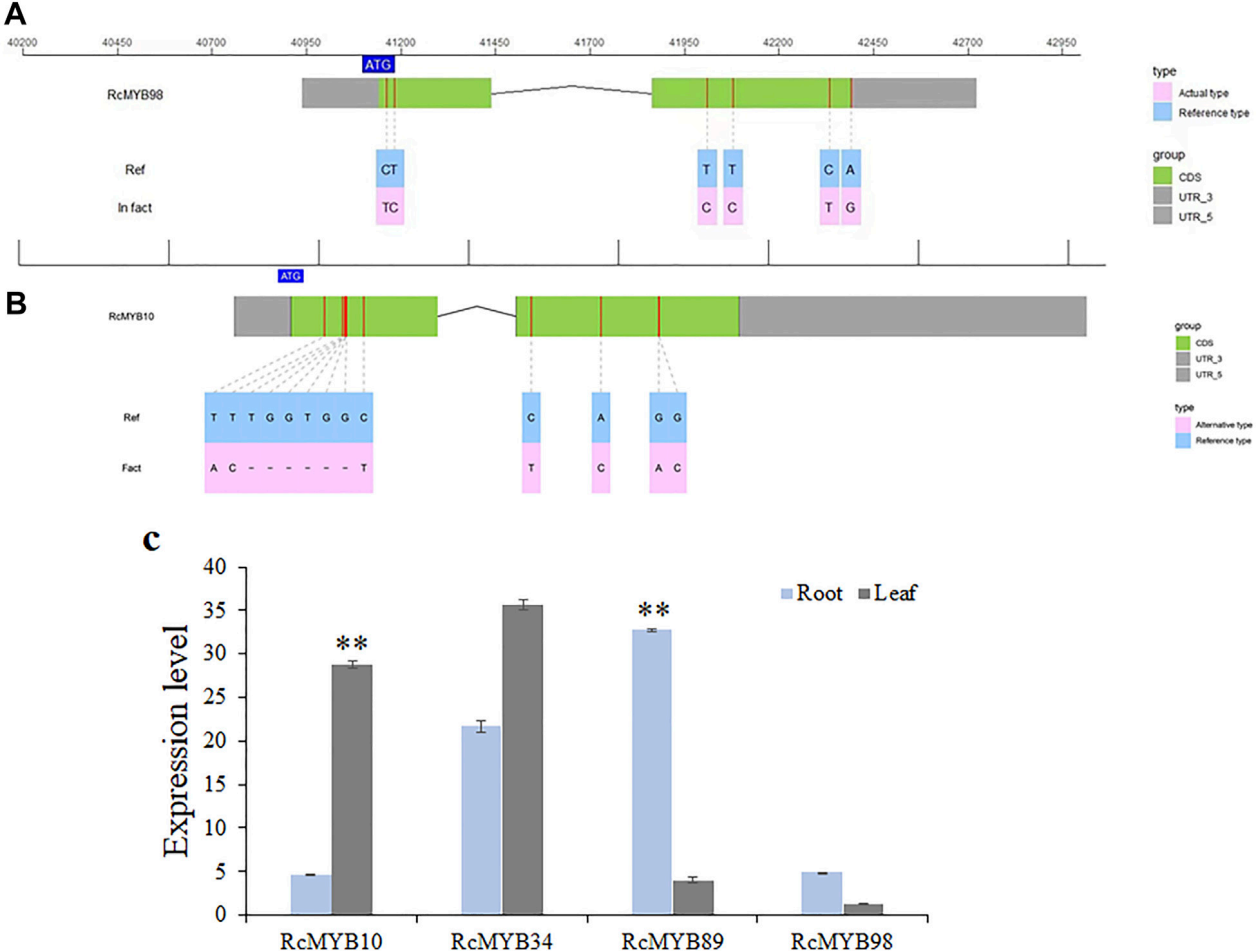
Gene cloning and expression level analysis of clustered RcMYBs. **(A)** Cloning and sequencing of *RcMYB98*. **(B)** Cloning and sequencing of *RcMYB10*. **(C)** Expression level analysis of *RcMYB10*, *RcMYB34*, *RcMYB89,* and *RcMYB98* in root and leaf. Values are the mean ± standard deviation of three biological replicates per treatment. “**” above each column indicates a significant difference (*p* < 0.01; n = 3).

To identify the accuracy of *RcMYB* genes and further experimental analysis, the genomic sequence and coding regions of *RcMYB10*, *RcMYB34*, *RcMYB89,* and *RcMYB98* were cloned and sequenced. It was found that the genomic sequence and coding regions of *RcMYB34* and *RcMYB89* showed a consistency with reference sequences (CCG031502.1 and CCG008637.1), whereas some single nucleotide polymorphisms (SNPs) were detected in the coding region of *RcMYB98* (Figure 6A). The SNPs in the coding region of *RcMYB98* only resulted in an amino acid change out of the R2R3 MYB domain. Besides, a 6 bp deletion and some SNPs were detected in the coding region of sequenced *RcMYB10*, resulted in three amino acids changed out of the R2R3 MYB domain (Figure 6B and Supplementary Figure S7).

Then, we analyzed the expression profiles of selected *RcMYB* genes in the root and leaf of *R. crenulata* by qRT-PCR (Figure 6C). As shown in Figure 6D, the expression level of *RcMYB34* in root and leaf was consistent, whereas the expression pattern of *RcMYB10*, *RcMYB89,* and *RcMYB98* was contrary. The expression level of *RcMYB10* in leaf was significantly higher than in root, but *RcMYB89* and *RcMYB98* expressed higher in root than leaf.

## Discussion

*R. crenulata* has broad application prospects in the field of modern medicine, and the second metabolites of its extract, such as flavonoids and glycosides, have been well-studied and utilized (Yousef et al., 2006; Yang et al., 2012; Zhou and Jiang, 2014). High altitude, with a lower oxygen and higher-pressure situation, contributed the most to the content of salidroside and other secondary metabolites in *R. crenulate*. Previous studies about the biosynthesis of secondary metabolites in *R. crenulate* usually pay more attention to the genes encoding the key enzyme of the synthesis pathway (Lan et al., 2013; Torrens-Spence et al., 2017), therefore ignoring the role of upstream regulation by transcription factors under abiotic stress conditions. In this research, the first systematic study of the transcription factor families in *R. crenulata* using bioinformatics tools and expression profiles based on the sequenced *R. crenulata* genome was performed. Here, a total of 139 *RcMYB* genes were identified in the *R. crenulata* genome, and their characteristics, gene structure, and conserved motifs were analyzed and predicted.

MYB transcription factor family members are widely involved in many biological processes in plants, such as plant growth, development, differentiation, response to abiotic and biotic stresses, and secondary metabolism (Ambawat et al., 2013; Liu et al., 2015). Whole-genome identification of MYB TFs has been conducted in many sequenced plants. Since its definitions in *Arabidopsis* (Stracke et al., 2001) and rice (Jiang et al., 2004b), *MYB* genes had been identified and cloned in many species, including grape (Matus et al., 2008; Fournier-Level et al., 2009), tobacco (Pattanaik et al., 2010), cauliflower (Chiu et al., 2010), soybean (Du et al., 2012), pear (Feng et al., 2015), *Casuarina equisetifolia* (Wang et al., 2021), and *Liriodendron chinense* (Wu et al., 2021). In this research, 101 R2R3-MYBs were identified, accounting for 72.6% of all RcMYBs (Supplementary Table S3). Most R2R3-MYB proteins are largely conserved and usually divided into the same subgroups as *Arabidopsis* MYB proteins, though, divergence between them exists. Comparative analysis of R2R3-MYBs from different plant species revealed that this TF family has experienced extensive expansion during evolution. The expansion of the MYB family in plants fits well with the results that many MYB members participate in various biological processes and in plant-specific processes. The MYB TFs were extensively duplicated during the process of plant evolution, leading to new members of the MYB TF family controlling specific functions. Previous studies in other plants confirmed that the expansion of the MYB multigene family was a result of genome-wide duplication or region-specific tandem duplication (Du et al., 2012; Lin and Rausher, 2021). In this study, a series of tandem gene duplications were observed in five scaffolds involving 10 *RcMYB* genes (Supplementary Figure S3), which suggested that tandem duplication events happened and played a role in the expansion of the large *RcMYB* family. Gene duplication is an important mechanism to generate new genes. A duplicate gene copy can lead to genetic and morphological diversification by evolving new gene functions (neo-functionalization), whereas the other copy can maintain the ancestral function (Rensing 2014). In this study, *RcMYB63* was closed to *RcMYB62* in the chromosome and considered as a duplicate gene copy, its C-terminal region contained motif 8 compared to *RcMYB62* (Figure 2 and Supplementary Figure S2). The same was observed between *RcMYB123* and *RcMYB124*, in which two motifs were identified in C- and N- terminal regions of *RcMYB124*. Besides, as a plant grown in a high-altitude and harsh environment, it seems that *R. crenulata* generates specific-function MYB genes during the process of adaptation to the environment and evolution. It was interesting that RcMYB85 contained a motif with 27 repeats and showed no alignment in *Arabidopsis* and rice. Although the phylogenetic analysis indicated that the homology of RcMYB85 and RcMYB137 was high and their motifs at N-terminal region were consistent, the repeated motifs at the C-terminal of RcMYB85 were unique (Figure 1 and Supplementary Figure S3). Further research about the function of RcMYB85 will be needed. It is interesting that this plant can survive in these extremely growing environments. The roles of *MYB* transcription factors in drought (Bi et al., 2016; Butt et al., 2017; Wang et al., 2017), heat (Zhao et al., 2017), cold (An et al., 2012; Yan et al., 2014; Yang et al., 2022), and other stress conditions (An et al., 2012; Guo et al., 2017; Liu et al., 2021) have been well-studied. In this research, the *cis*-acting elements of the upstream sequence of the *RcMYB* genes were analyzed, and the results showed that in addition to numerous score promoter elements, there were indeed many environmental response elements and hormone response elements, such as the light-responsive elements GT1-motif, abscisic acid responsiveness element ABRE, MeJA-responsiveness TGACG-motif, auxin-responsive element TGA-motif, and anaerobic induced response element ARE (Figure 4 and Supplementary Table S4). These motifs indicated that the expression of *RcMYB* genes would be induced by many abiotic factors to enhance or inhibit transcription. Besides, there were MYB binding sites involved in some elements like MRE, which suggested some *RcMYBs* could cooperate with other *RcMYBs* to perform or emphasize functions. These *cis*-elements might be helpful for the adaptation of *R. crenulata* to the tough environment.

The gene expression patterns can provide important clues to explore gene function. In this research, WGCNA analysis indicated that *RcMYB10* and *RcMYB34* correlated with the genes encoding key enzymes involved in the biosynthesis of salidroside. The distribution of second metabolites was different in root and leaf of *R. crenulata*. Although *RcMYB10*, *RcMYB34*, *RcMYB89,* and *RcMYB98* were predicted to be involved in the secondary metabolism process, their regulation of secondary metabolites was probably varied.

In this study, the MYB transcription factor family in *R. crenulata* was identified, and a series of bioinformatics analyses were carried out to reveal the characteristics of *RcMYBs*. Further, *RcMYB* members that may be involved in the secondary metabolic process of *R. crenulata* were cloned and analyzed. After sequencing and analysis, the understanding of its function and structure increased, and information such as *cis*-acting elements and target genes were predicted. This research has provided the data basis for further research on MYB transcription factors of *R. crenulata*.

## Data Availability

The datasets presented in this study can be found in online repositories. The names of the repository/repositories and accession number(s) can be found in the article/Supplementary Material.
