# Supplementary figures and images for: Genome-wide Identification and Expression Analysis of RcMYB Genes in Rhodiola crenulata
Source: Front Genet. 2022 Mar 31;13:831611. doi: 10.3389/fgene.2022.831611 (PMC9008588; doi:10.3389/fgene.2022.831611)

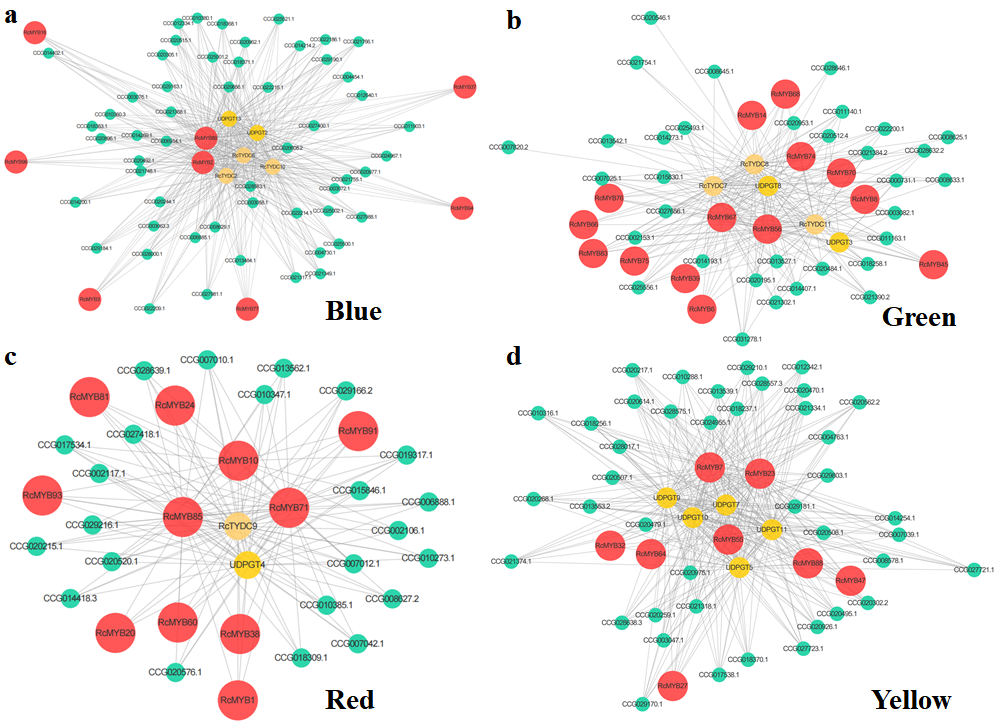

Supplement: Supplementary file 1 [file Image6.TIF]

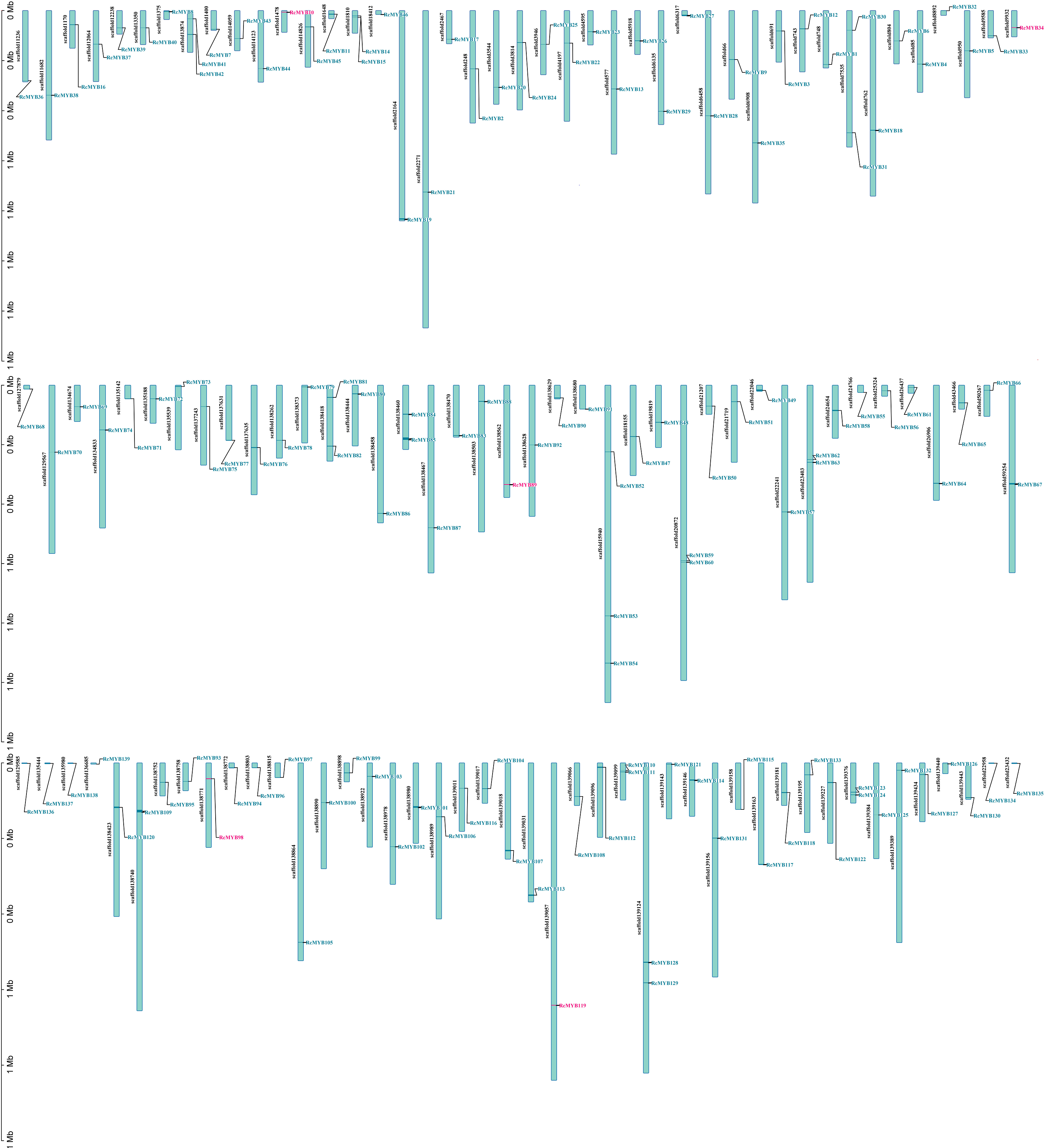

Supplement: Supplementary file 2 [file Image3.TIF]

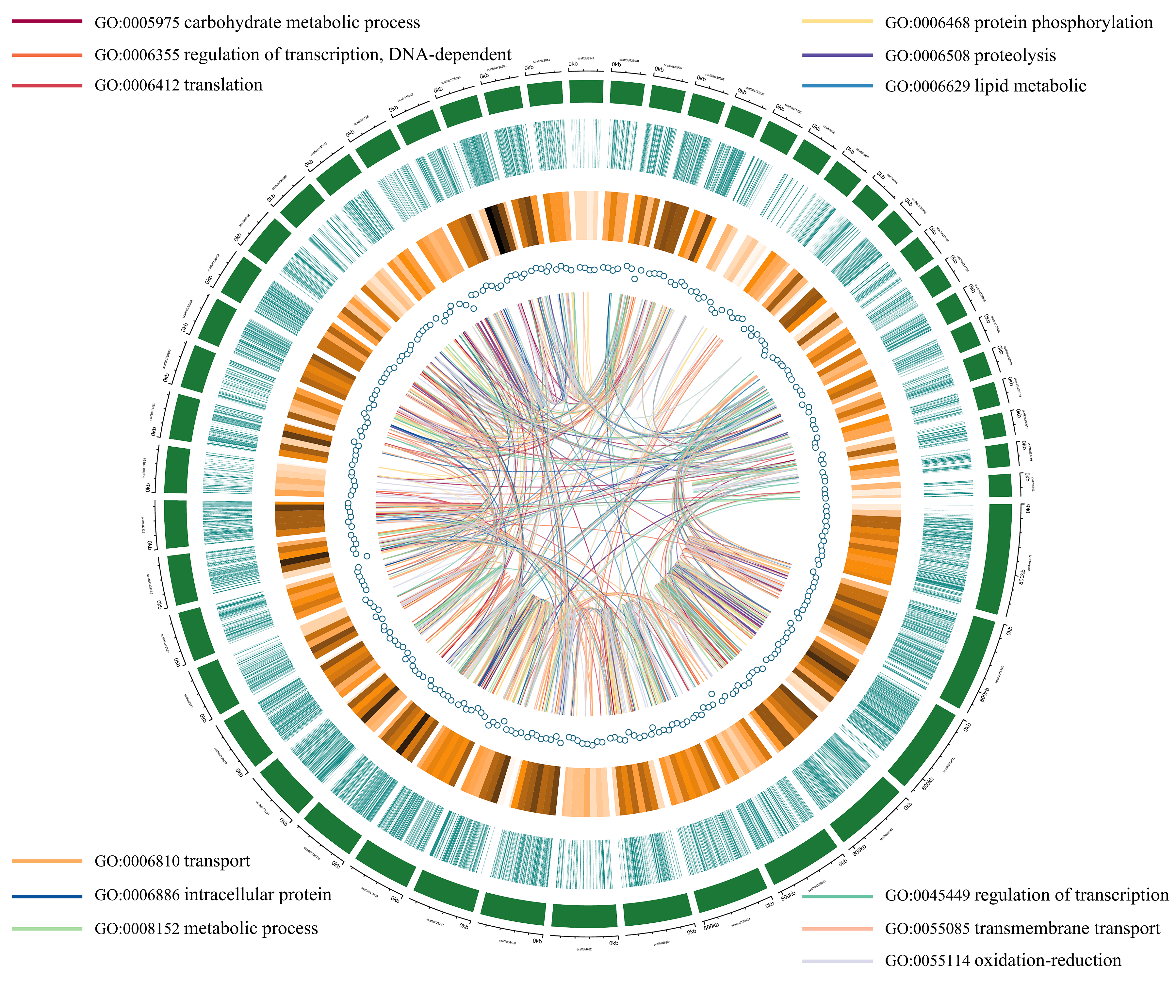

Supplement: Supplementary file 3 [file Image4.TIF]

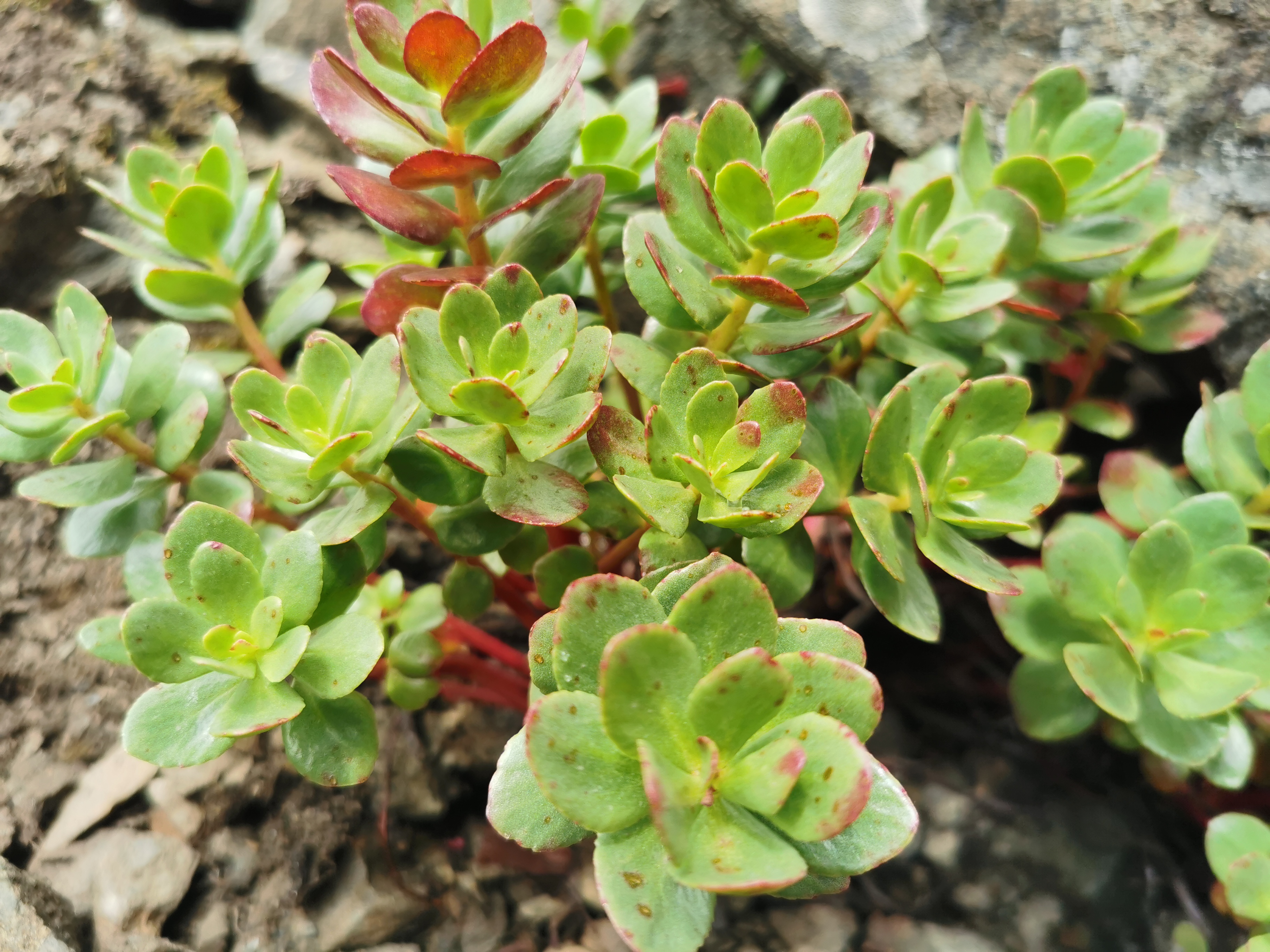

Supplement: Supplementary file 4 [file Image1.JPEG]

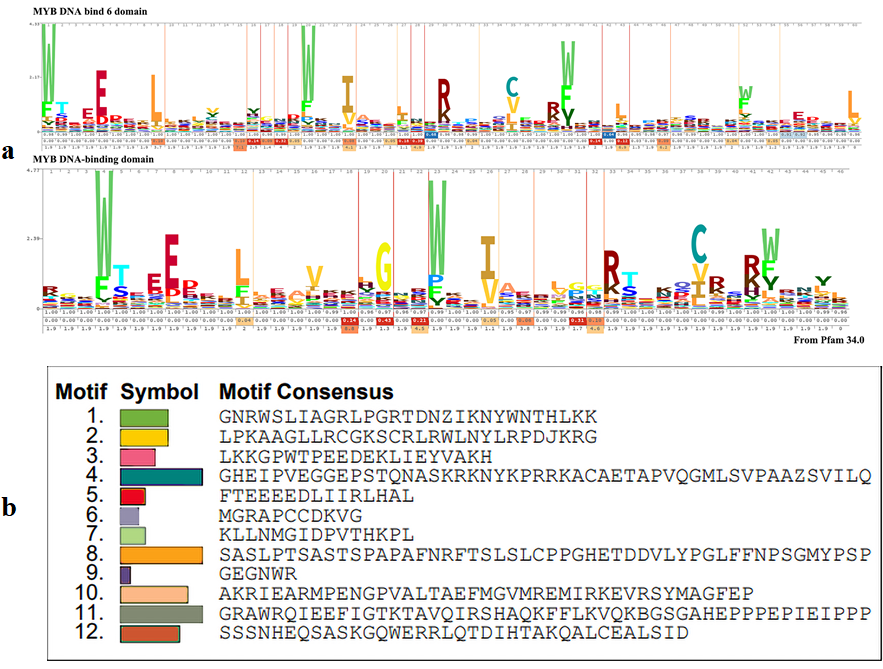

Supplement: Supplementary file 5 [file Image2.TIF]

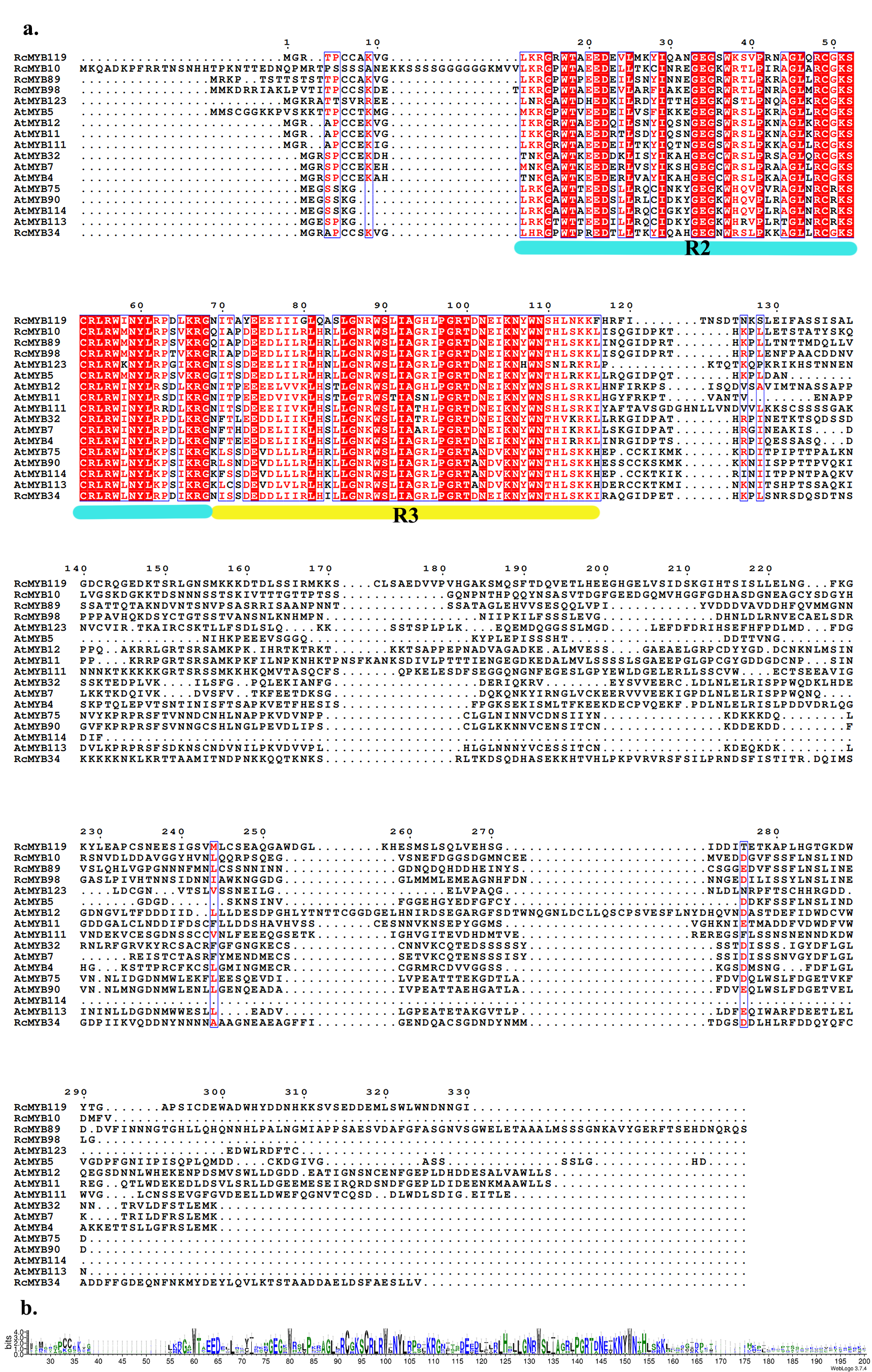

Supplement: Supplementary file 6 [file Image7.TIF]

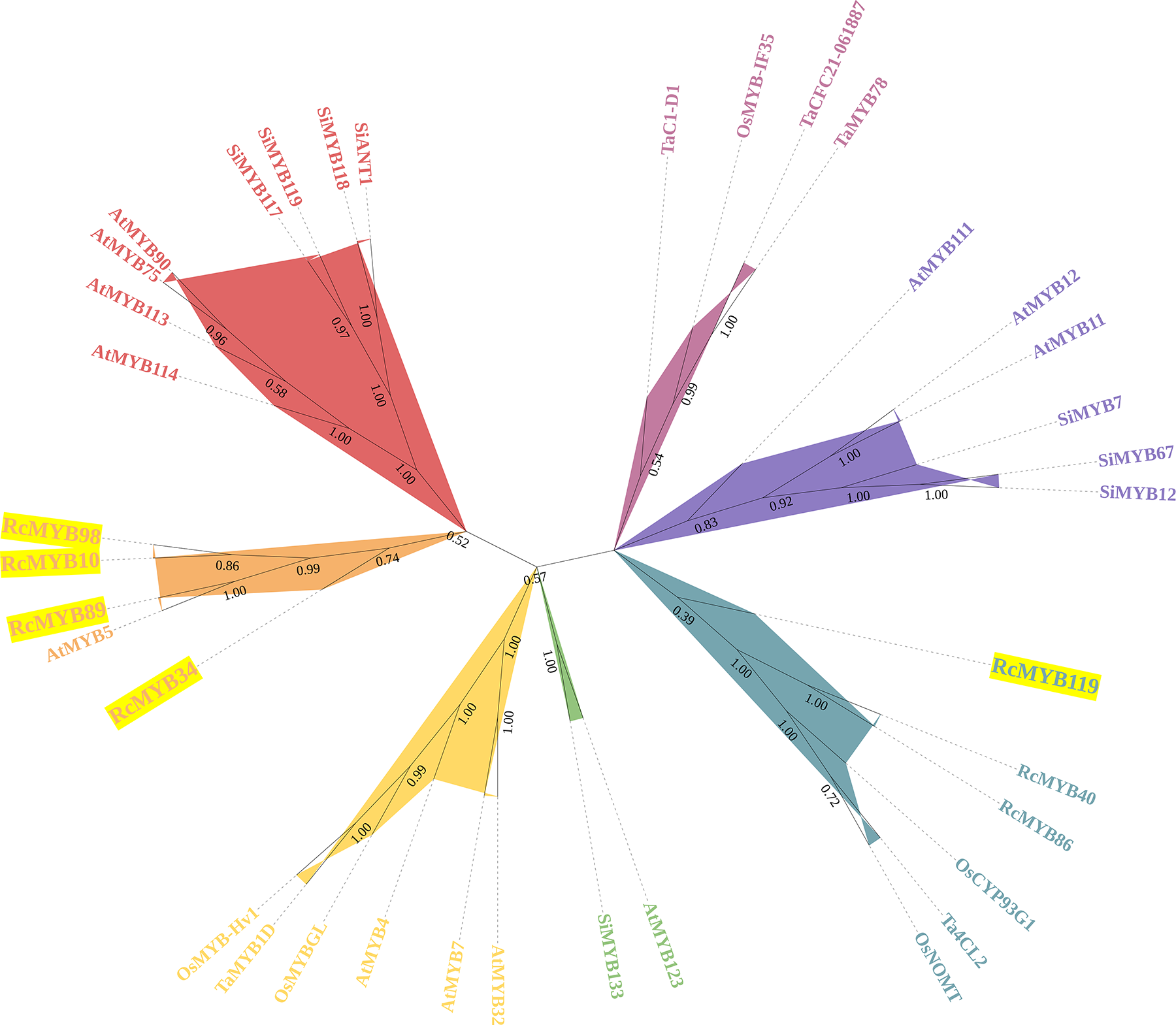

Supplement: Supplementary file 11 [file Image5.TIF]
