# Supplementary material for: Genome-wide Identification and Expression Analysis of RcMYB Genes in Rhodiola crenulata
Source: Front Genet. 2022 Mar 31;13:831611. doi: 10.3389/fgene.2022.831611 (PMC9008588; doi:10.3389/fgene.2022.831611)
Supplement: Supplementary file 7 [file Table2.DOCX]

**Supplementary Table 2 Primers used in this study.**

|  | Forward 5’-3’ | Reverse 5’-3’ |
| --- | --- | --- |
| Primers used in vector construction and sequencing | | |
| MYB10-CDNA | ATGAAACAAGCAGACAAGCCTTT | TACAAACATGTCATCATTGATCAAAGA |
| MYB34- CDNA | ATGGGGCGAGCTCCTTGT | AACTAACAAGGACTCGGCGAAG |
| MYB89- CDNA | ATGAGGAAGCCAACGTCAACG | TCAACTTTGCCTCTGATTATCATGC |
| MYB98- CDNA | ATGATGAAGGATCGGAGGATCG | TCCTAGCTCGTTGATCAGAGAGTTC |
| MYB119- CDNA | ATGGGAAGGACGCCATGTTG | AATCCCATTATTATCATTCCACAACC |
| Primers used in analysis of gene expression pattern | | |
| Myb10-qpcr | CATAAACAGGGAGGGAGAG | GCTGAGGATGTGTATTAGGG |
| Myb34-qpcr | GCGGTGACCCGATTATCAAAGT | ACATTTTGTTGAAGTTCTGCTC |
| Myb89-qpcr | ATTATCTCCGCCCATCTGTCAA | TCTTACTCAGATGCGTGTTCCA |
| Myb98-qpcr | CCTCCTCCAGCAGTTCACCAGA | ATCAATGCTGTTGTTTGTATGC |
| 18S-rRNA | ATGATAACTCGACGGATCGC | CTTGGATGTGGTAGCCGTTT |

The sequence of 18S-rRNA was cited from Reference article (Lan et al., 2013).
