# Supplementary material for: Genome-wide Identification and Expression Analysis of RcMYB Genes in Rhodiola crenulata
Source: Front Genet. 2022 Mar 31;13:831611. doi: 10.3389/fgene.2022.831611 (PMC9008588; doi:10.3389/fgene.2022.831611)
Supplement: Supplementary file 9 [file Table3.DOCX]

**Supplementary Table 3** The categories of *RcMYBs* based on their number of MYB domain.

| **Category** | **Gene ID** |
| --- | --- |
| 1R-MYB | RcMYB11, RcMYB12, RcMYB18, RcMYB19, RcMYB31, RcMYB32, RcMYB33, RcMYB35, RcMYB40, RcMYB44, RcMYB48, RcMYB49, RcMYB56, RcMYB70, RcMYB76, RcMYB82, RcMYB84, RcMYB86, RcMYB88, RcMYB92, RcMYB96, RcMYB100, RcMYB101, RcMYB110, RcMYB116, RcMYB118, RcMYB123, RcMYB124, RcMYB125, RcMYB126, RcMYB131, RcMYB135, RcMYB138, RcMYB139 |
| R2R3-MYB | RcMYB1, RcMYB2, RcMYB3, RcMYB4, RcMYB5, RcMYB6, RcMYB7, RcMYB8, RcMYB9, RcMYB10, RcMYB13, RcMYB14, RcMYB15, RcMYB16, RcMYB17, RcMYB20, RcMYB21, RcMYB22, RcMYB23, RcMYB24, RcMYB25, RcMYB26, RcMYB28, RcMYB29, RcMYB30, RcMYB34, RcMYB36, RcMYB37, RcMYB38, RcMYB39, RcMYB41, RcMYB42, RcMYB43, RcMYB45, RcMYB46, RcMYB47, RcMYB50, RcMYB51, RcMYB52, RcMYB53, RcMYB54, RcMYB55, RcMYB57, RcMYB59, RcMYB60, RcMYB61, RcMYB62, RcMYB63, RcMYB64, RcMYB65, RcMYB66, RcMYB67, RcMYB68, RcMYB69, RcMYB71, RcMYB72, RcMYB73, RcMYB74, RcMYB75, RcMYB77, RcMYB78, RcMYB79, RcMYB80, RcMYB81, RcMYB83, RcMYB87, RcMYB89, RcMYB90, RcMYB91, RcMYB93, RcMYB94, RcMYB95, RcMYB97, RcMYB98, RcMYB99, RcMYB102, RcMYB103, RcMYB104, RcMYB105  RcMYB106, RcMYB107, RcMYB108, RcMYB109, RcMYB111, RcMYB112, RcMYB113, RcMYB114, RcMYB115, RcMYB117, RcMYB119, RcMYB120, RcMYB121, RcMYB122, RcMYB127, RcMYB129, RcMYB130, RcMYB132, RcMYB133, RcMYB134, RcMYB136, RcMYB137 |
| 3R-MYB | RcMYB27, RcMYB58, RcMYB85, RcMYB128 |
